# A rare cause of dyspnea and cough: Diffuse tracheal papillomatosis

**DOI:** 10.1590/0037-8682-0109-2022

**Published:** 2022-07-25

**Authors:** Veysel Ayyildiz, Yener Aydin, Hayri Ogul

**Affiliations:** 1 Suleyman Demirel University, Medical Faculty, Department of Radiology, Isparta, Turkey.; 2 Ataturk University, Medical Faculty, Department of Thoracic Surgery, Erzurum, Turkey.; 3 Duzce University, Medical Faculty, Department of Radiology, Duzce, Turkey.

**FIGURE 1:**
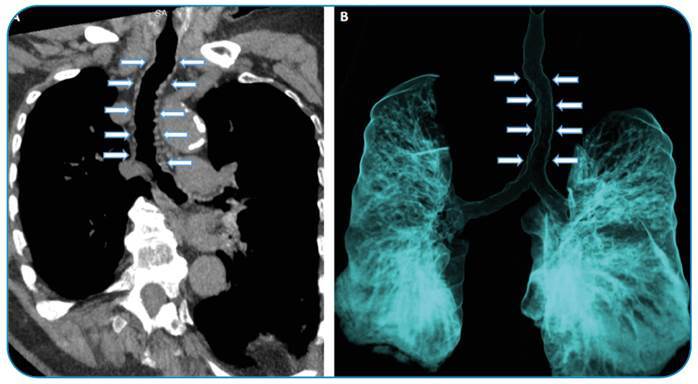
Chest computed tomography findings in the coronal **(A)** and air-specific reformat **(B)** views demonstrating diffuse tracheal papillomatosis (arrows).

An 85-year-old man presented to our hospital for the evaluation of dyspnea and cough. Diffuse tracheal papillomatosis was noted on computed tomography findings (Figure 1A and Figure 1B).

Respiratory papillomatosis usually develops from persistent infection of the respiratory mucosal epithelium by human papillomavirus 6 or 11 strains. Approximately 5% of patients with laryngeal papillomatosis have involvement of the trachea, proximal bronchi, or both. Malignant transformation rarely occurs in cases of recurrent respiratory papillomatosis^1^ ^,^ ^2^. Tracheal papillomatosis should be kept in mind as a rare cause of cough and dyspnea.
